# Ventral Hippocampus Modulates Prefrontal Control of Background Contextual Fear After Cued Extinction

**DOI:** 10.1111/ejn.70287

**Published:** 2025-10-23

**Authors:** Marco N. Pompili, Adam Eckmier, Ralitsa Todorova, Margot Tirole, Bill P. Godsil, Thérèse M. Jay

**Affiliations:** ^1^ Institut de Psychiatrie et Neurosciences de Paris (IPNP), INSERM U1266, CNRS GDR3557 Université Paris Cité Paris France; ^2^ Centre Interdisciplinaire de Recherche en Biologie (CIRB) ‐ CNRS UMR 7241 ‐ INSERM U1050, Collège de France, Université PSL Paris France; ^3^ Institut de Neurosciences des Systèmes, INSERM UMR1106 Aix‐Marseille Université Marseille France

**Keywords:** aversive conditioning, contextual modulation, hippocampus, optogenetics, prefrontal cortex

## Abstract

Understanding the neural mechanisms underlying fear extinction is crucial for improving anxiety disorder treatments. Previous studies have shown that activity in the ventral hippocampus (vHPC) to prelimbic cortex (PL) pathway regulates conditioned fear towards discrete cues after extinction. However, its role in contextual fear was unclear. In this study, we used optogenetics in rats to selectively stimulate vHPC terminals in the PL, which in anaesthetized animals led to responses in approximately 12% of PL neurons. In freely behaving animals, stimulating vHPC–PL projections during baseline context exposure, prior to the presentation of any discrete cue in post‐extinction fear renewal tests, decreased contextual fear without affecting motricity or contextual fear immediately after conditioning. These results indicate that vHPC‐PL projections modulate contextual fear expression after extinction. While task‐specific features may contribute to the observed effect, our study fills a gap in understanding how the interaction between the vHPC and the mPFC regulates fear expression when contextual recall is followed by an extinction session.

## Graphical abstract

Previous studies showed that ventral hippocampus (vHPC) projections to prelimbic cortex (PL) regulate fear towards discrete cues after extinction, but their role in contextual fear was unclear. Using optogenetics in rats, we stimulated vHPC terminals in PL during baseline context exposure before cue presentation in post‐extinction tests. Stimulation reduced contextual fear without affecting motricity or fear immediately after conditioning, indicating that vHPC‐PL projections modulate contextual fear expression following extinction.

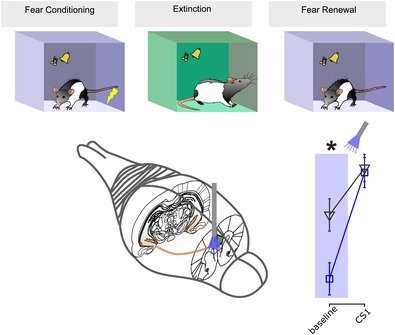

**Abbreviations**
ChR2: channelrhodopsin2
CS: conditioned stimulus
EBT: exposure‐based therapies
GFP: green fluorescent protein
HPC: hippocampus
mPFC: medial prefrontal cortex
PL: prelimbic cortex
vHPC: ventral hippocampus
US: unconditioned stimulus

## 1 Introduction

Traumatic memories can help anticipate future threats but can also lead to anxiety disorders, affecting 25% of the population that has experienced a trauma (Ressler et al. 2022). Extinction of these fear responses is crucial for treating anxiety, often through exposure‐based therapies (EBTs; Dunsmoor et al. 2015). However, EBTs are context‐dependent, meaning that fear extinction learned in therapy may not generalize to new settings, leading to relapse when triggers are encountered in different environments (Vervliet et al. 2013).

This context‐dependency can be modelled in rodents using paradigms where pavlovian fear conditioning is followed by extinction training and then a fear renewal test. A conditioned stimulus (CS), for example an auditory stimulus (e.g., a tone), when paired with an aversive stimulus (e.g., a mild electric foot shock), triggers a fear response. During extinction, conditioned fear gradually diminishes with repeated presentations of the CS in isolation. However, this reduction is context‐specific, and fear responses may re‐emerge in a context different from where extinction occurred—a phenomenon known as fear renewal (Bouton et al. 2006).

The hippocampus (HPC) and medial prefrontal cortex (mPFC) play key roles in this process (Goode et al. 2018). The HPC, essential for contextual memory (Maren et al. 2013), projects to the mPFC (Jay and Witter 1991), influencing emotional and fear regulation with potentially a crucial role in the pathophysiology of several psychiatric disorders (Godsil et al. 2013). Indeed, evidence suggests that the vHPC to mPFC pathway is implicated in regulating fear behaviour. Specifically, the activity of the connections from the vHPC to the dorsal mPFC, the prelimbic cortex (PL), modulates the expression of innate (Padilla‐Coreano et al. 2016) and learned fear (Vasquez et al. 2019; Szadzinska et al. 2021). Indeed, pharmacogenetic activation of vHPC cells projecting to PL after extinction training attenuates subsequent cued fear renewal (Vasquez et al. 2019). Consistently, optogenetic stimulation of these neurons' terminals during CS presentations after extensive extinction decreases cued fear (Szadzinska et al. 2021).

These results suggest that vHPC‐PL projections regulate learned cued fear after extinction. What remains unexplored is whether, after extinction, vHPC inputs to the PL also regulate contextual fear. To investigate this, we used optogenetics to control the activity of vHPC terminal projections in the PL. We stimulated these projections during baseline exploration of the testing chamber in a context‐dependent fear renewal test, which did not affect subsequent cued fear expression but reduced contextual fear. Surprisingly, contextual fear was unaffected by stimulation of vHPC terminals in the PL during a contextual fear conditioning recall test, suggesting that these projections specifically gate contextual fear after cued fear extinction has occurred.

## 2 Materials and Methods

### 2.1 Animals

We used 162 male Long‐Evans rats (260–340 g at the time of surgery, 2–4 months old), which were housed in groups of 3 or 4 before surgery and then individually. They were maintained at about 21°C in a well‐ventilated room with a light/dark cycle of 12 h with free access to food and water. Upon arrival in the lab, the animals were allowed at least 3 days of acclimation before being handled daily by the experimenter for at least 2 days prior to surgery. Twenty‐two and forty‐five animals were used for the first and second fear renewal experiments, respectively. Thirty‐six and forty‐nine animals were used for the context freezing and ambulatory distance experiments, respectively. Ten animals were used for the acute electrophysiology recordings. Rats were assigned to the different groups randomly. The target sample sizes were established based on previous studies (e.g., Kim et al. 2016; Szadzinska et al. 2021) and practical considerations, and the sample sizes indicated in the figures correspond to the animals retained for the analyses after those without good expression of the viral construct, with misplacement of optic fibres/electrodes, or not displaying satisfactory extinction were discarded (see Sections 2.5 and 2.9). All the experimental procedures were performed in accordance with institutional guidelines and the French national and European laws and policies and approved by Paris Descartes University Ethical Committee.

### 2.2 Virus Injection and Chronic Fibre Implantation

To selectively manipulate the projection from the ventral hippocampus (vHPC) to the prelimbic cortex (PL), we used an optogenetic approach. One hundred eight animals were injected in the vHPC with AAV9‐CAG‐ChR2‐GFP, an adeno‐associated virus carrying channelrhodopsin 2 (ChR2) and tagged with a green fluorescent protein (GFP) (University of North Carolina Vector Core, USA), while 54 animals received the injection of an AAV9‐CAG‐GFP control virus. Rats were anaesthetized with an intraperitoneal injection of a ketamine/xylazine mix (90 and 15 mg/kg, respectively) and subcutaneously with buprenorphine hydrochloride. All animals received unilateral injections to allow photic stimulation via a single‐channel optical commutator during behaviour, except for those used for the electrophysiology recordings who were injected bilaterally to maximize the number of recorded cells per animal. For each injection, 0.8 μL of solution containing the virus was pressure‐injected into the vHPC (−6 mm AP, ±5.4 mm ML, 6 mm DV from dura mater) at a rate of 0.1 μL/min. The tissue was allowed to recover for 5 min before needle retraction.

For the 10 animals used for a control experiment with acute electrophysiology recordings (see below), the scalp was then sutured, and the animals were placed in their home cage on a heating pad until they woke up. The other animals instead also received the implantation of light delivery cannulas in PL during the same surgery, to allow light delivery specifically at vHPC axon terminals within the PL during behavioural tasks. These animals received a craniotomy above the mPFC (+3.2 mm AP/0.8 mm ML of bregma), and a single mono‐fibre optic cannula (Doric Lenses) was positioned in the PL with the lower edge of the cannula receptacle positioned flush with the skull surface. Two anchoring screws were installed at 26.7 mm AP/5.4 mm ML to bregma and 24.0 mm AP/3.0 mm ML to bregma. Next, bonding adhesive (Superbond L‐type polymer, Sun Medical) was applied to the skull surface before a series of layers of self‐curing composite dental resin (Dentalon Plus, Farbe, France) was built up over the skull until a cap sufficient to hold the cannula in place formed. Rats were left to recover for 6 weeks post‐injection in order to obtain robust viral expression in the vHPC axon terminals in mPFC. Rats were handled four times per day for 3 days prior to any behavioural testing. Handling involved briefly removing the rat from its cage while manipulating its cannula's dust cap.

### 2.3 Control Electrophysiological Acute Experiment

To confirm that optogenetic stimulation of vHPC terminals in the PL effectively modulates neuronal activity there, a subset of rats (*n* = 6) was anaesthetized with isoflurane in oxygen (5%) and then maintained with an IP injection of urethane (1.7 g/kg), as urethane is known to preserve natural cortical activity patterns, including up and down states (see Clement et al. 2008). The rat was then placed in the stereotaxic frame, the skull exposed, and craniotomies were drilled above PL (a 4 × 2 mm area above the right and left PL cortices; see below for the exact coordinates of implantation of the optrode) and above the cerebellum to place the reference electrode. Custom‐made optrodes recorded and stimulated bilaterally brain activity. They consisted of two 200 μm diameter optic fibres placed 1.6 mm apart and surrounded by four octrodes made of twisted tungsten wire, cut 500 μm below the tip of the fibres. The electrodes were gold plated to reduce impedance to about ±100 kΩ. The implant was positioned so that the fibres were placed at ±0.8 mm ML; +3 mm AP. It was then very slowly lowered in the brain until PL was reached and suitable spike activity was detected on both sides. At each site, the optic stimulation protocol was applied, and afterwards, the implant was lowered a few tens of microns to find new cells, until the ventral limit of PL.

**FIGURE 1:**
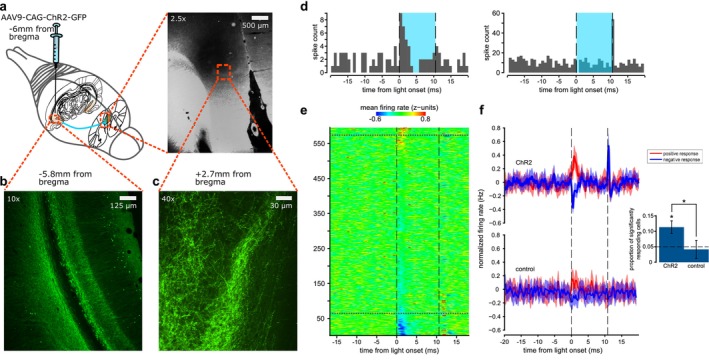
PL neural responses to the stimulation of vHPC terminals expressing ChR2. (a) Schematic depicting the strategy used to obtain ChR2 expression in vHF efferents to PL with the unilateral injection site of the AAV9‐CAG‐ChR2‐GFP construction in the vHPC and the projection of infected neurons to the PL. (b) ChR2 expression in vHPC CA1 (labelled by GFP). (c) GFP‐labelled ChR2 expression in vHPC axon terminals in PL (bottom) and microphotography of the mPFC depicting the site of the greater magnification (top). (d) Peri‐event time histograms for example cells with excitatory (left) and inhibitory‐rebound (right) responses. (e) *z*‐score average firing rate of all recorded PL units in ChR2 expressing animals around light pulses stimulation. About 12% displayed excitatory or inhibitory responses to laser onset. Vertical dashed lines depict the beginning and end of the light pulses. Horizontal dashed lines segregate units which displayed a significant response (see Section 2) within 12 ms from the light pulse onset. (f) Firing rate evolution around light stimuli for significantly excited (red) and inhibited (blue) cells in ChR2 (*n* = 4, left) and control (*n* = 2, right) animals. Inset, proportion of significantly responding units as assessed with the jitter analysis (see Section 2), *p* < 0.05, Sign rank test. *p* < 0.05.

Brain activity was recorded with a 64‐channel analogue recording system with Cheetah software control (Neuralynx, USA). Signals were differentially amplified 1000 times, bandpass filtered between 0.1 and 6000 Hz, and digitized at 32,556 kHz. The differential reference was manually selected from a bipolar electrode implanted in the cerebellum. A large metallic box was placed above and around the stereotaxic frame to act as a Faraday cage, protecting the implant and the pre‐amplifiers from noise. Optogenetic stimulations were controlled with a Power 1401 interface and Spike2 software (CED, UK). Light was delivered with an MLD Laser Diode Module 473 nm laser (Cobolt, Germany), and the light output was split into two beams of equal power for each hemisphere with a minicube splitter (Doric Lenses, Canada). We used 20 Hz train‐pulse stimulations of four different durations (0.5, 1, 5, and 15 s) at different light intensities (6.7 mW and 11 mW). The latter near‐saturation power was equivalent to the one used for the behavioural experiment, while the former was used in the eventuality of near‐saturation power hiding neural responses. While the goal of the behavioural experiment was to induce a cognitive or behavioural effect, the purpose of the electrophysiological experiment was to verify that 20 Hz stimulation of vHPC terminals in mPFC induces firing responses in mPFC neurons. Since this control experiment was exploratory, we used a variety of stimulation parameters, ranging from 0.5 to 15 s for the duration of the trains of pulses and from 6.7 to 11 mW intensities. No qualitative difference was found between different pulse parameters, so data were pooled for the neuronal responses analyses. The fact that even such short trains of pulses were sufficient to induce a downstream response ensures that longer stimulations, such as those employed in the behavioural experiment, were also effective. We obtained neural responses with both intensities and with trains of all durations, and therefore all data were pooled to produce the plots of Figure 1. Each trial consisted of a 5 min baseline recording followed by the 4 durations of train pulses randomly presented with an inter‐train interval of 30 s. Neurophysiological data were processed with the NeuroSuite software as described in detail by Hazan et al. 2006. In brief, to extract spiking activity, wide‐band signals were high‐pass filtered (with a non‐linear median‐based filter, cutoff 300 Hz) and thresholded (at 1.3 *z*‐score) using NDManager plugins (see https://neurosuite.sourceforge.net/). Extracted spike waveforms were sorted via a semi‐automatic cluster cutting procedure using KlustaKwik (Harris et al. 2000; Shabnam et al. 2014; https://klustakwik.sourceforge.net/) and Klusters (Hazan et al. 2006). Neurophysiological and stimulation data were explored using NeuroScope (Hazan et al. 2006). The waveform, autocorrelogram, and raw traces of each cluster discriminated by the programme were carefully examined, and only those showing low noise were retained for further analyses.

All further analysis aimed at testing whether the well‐isolated single units responded to the optogenetic stimulations was performed using the FMA toolbox (http://fmatoolbox.sourceforge.net) and custom‐written programmes in Matlab (Mathworks, USA). We categorized cells as responsive or not responsive based on a jitter method (as in Reimer and Hatsopoulos 2010). In order to create a null hypothesis distribution of the data relative to the stimulations, we first created surrogate spiking data. The timestamps of spikes around the pulses of light were jittered in time by adding a random shift extracted from a uniform distribution (ranging between 1 and 10 ms). The resulting surrogate histograms (100 iterations) were then compared with the original non‐jittered one. Cells with at least one bin (1 ms) exceeding the 99th percentile of the surrogate distribution (*p* ≤ 0.01) within 12 ms from the pulse onset were considered responsive. The 12 ms window was chosen to capture responses to pulse onset (at 0 ms) and offset (at 10 ms). Monosynaptic responses are expected to occur within approximately 2 ms following stimulation of presynaptic terminals (Jouhanneau et al. 2015), while monosynaptic rebound firing may occur shortly after light offset. To estimate the proportion of responsive units that could be expected by chance using the jitter method, we generated control data by shuffling the spike times across the entire recording. Specifically, for each unit, we randomized the order of inter‐spike intervals, thereby preserving the unit's natural firing patterns and burst properties while disrupting the temporal alignment of spikes relative to the experiment. We then applied the same jitter analysis to this shuffled dataset, yielding an estimate of the chance level for detecting responsiveness.

### 2.4 Fear Conditioning Apparatuses and Data Processing

We conducted a series of behavioural experiments to determine the role of the vHPC‐PL pathway in contextual fear after extinction. All training and test sessions were conducted in a standard conditioning chamber kept inside a sound‐attenuating cubicle (VCF‐007, Med Associates, USA). This chamber had the internal dimensions of 30 × 24 × 33 cm, with aluminium sidewalls, an interchangeable rear wall, and a transparent Plexiglas door. The grid floor was connected to a constant current scrambled shock generator (ENV‐414, Med Associates, USA), which delivered the US footshock (2 s, 0.6 mA). The rats were placed in the chamber individually and were connected to a patch‐cable/rotary‐joint/laser assembly that was controlled by Med Associates hardware and software. Three configurations of this chamber were used in the conditioning experiments: α, β, and γ. In configuration α, the chamber was dark, except for two infrared light sources located above the training box, the rear panel was constituted by a transparent Plexiglas wall, and ~0.2 mL of a mint‐scented cleaning solution (Simple Green, Sunshine Makers) was put in a pan underneath the grid floor. Configuration β was the same darkened chamber as α, but was scented with 1% acetic acid in the collection pan, and had white plastic floor and curved wall inserts. Configuration γ had the same floor and wall plastic inserts as β, but it was scented with 1% ammonia; the lighting was on, as well as a ventilation fan. A well‐trained experimenter who was blind to both the animal's group (ChR or control) and the session type used an instantaneous time‐sampling procedure to characterize behavioural freezing from the video files. Freezing was defined as the absence of movement except for breathing.

The light source used in all behavioural experiments was blue light (473 nm) from a laser (BL473T3‐100FC, Shanghai Laser) through the patch cable/rotary joint assembly and into the fibre optic cannula with a light power output measured at the fibre tip of 11 mW. The light was delivered as light pulses at 20 Hz.

### 2.5 Fear Renewal Protocols

In the fear renewal protocols, the rats underwent auditory fear conditioning in context A (chamber configuration α) for 12 min during which they received three 30 s presentations of the auditory CS (2 KHz pure tone) that co‐terminated with the US at 300, 512, and 724 s after the initiation of the control programme, which was activated immediately following the placement of the animal in the chamber. Extinction training started the following day with daily 25‐min sessions with 24 CS presentations starting at 300 s after the initiation of the session separated by 20 s intervals. For each rat, extinction training continued until the average time spent freezing across the first four CS presentations was below 60%. The animals that did not reach this threshold by the seventh day of extinction training were excluded from further analysis.

**FIGURE 2:**
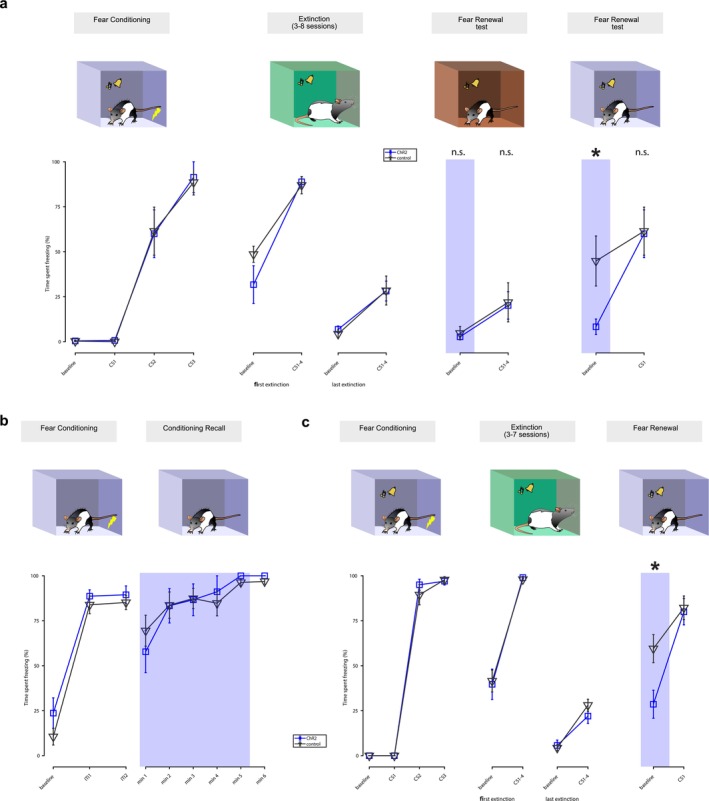
vHPC‐PL pathway stimulation effects on contextual fear expression. (a) Top, experimental design of the compound ABCA fear renewal protocol. Optogenetic stimulation of the vHPC‐PL pathway took place during the baseline pre‐CS presentation periods in both renewal test sessions. All sessions took place on different days. Bottom, comparison of the average time spent freezing during the different learning stages of the protocol for the stimulated animals (ChR2, *n* = 7) and controls (*n* = 7). Whiskers depict ±SEM, the shaded blue rectangle indicates when laser was ON. [n.s.: no significant difference between groups; *: *t*(12) = 2.51, *p* = 0.027; unpaired *t*‐test, see Section 2 and Data S1]. (b) Top, experimental design of the contextual fear conditioning and recall test protocol. Bottom, comparison of percentage of time spent freezing during the baseline and the inter‐trial intervals (ITI) of the contextual fear conditioning session (left) and during the 6 min of the contextual fear recall test (right) for the laser stimulated group (*n* = 9) and control animals (*n* = 19). Whiskers depict ±SEM, the shaded blue rectangle indicates when laser was ON. [No significant difference at any time point; unpaired *t*‐test, see Section 2 and Data S1]. (c) Top, experimental design of the ABA fear renewal protocol. Below, comparison of the average time spent freezing during the different learning stages of the protocol for the stimulated animals (ChR2, *n* = 10) and controls (*n* = 18). Same format as panels (a) and (b). CS 1–4 = average freezing over the first four CS presentations. [*: *t*(24) = 2.80, *p* = 0.010; unpaired *t*‐test, see Section 2 and Data S1]

In the compound ABCA design (Figure 2a), all extinction sessions took place in context B (chamber configuration β). The day after the last extinction training, the first fear renewal test took place in context C (chamber configuration γ). The following day, a second fear renewal test took place in the same context of fear conditioning, A. During the fear renewal tests, rats were placed in the chamber for 6 min, where after a 300 s baseline, a CS was presented. For the 10 animals that received the injection of the control GFP virus and the 12 that received the injection of the ChR2 carrying virus, light stimulation was on for 300 s (with 20 Hz pulses) during the baseline before the CS presentation.

In the ABA design (Figure 2c), we controlled for a possible effect of the specific extinction context by using two configurations for context B that were chamber configuration β for half of the animals and chamber configuration γ for the other half. In parallel with extinction training, each day, the rats underwent a context exposure session during which they were placed for 25 min in context C (which was chamber configuration γ for those undergoing extinction training in β, and β for the others) after or before auditory extinction (the order of extinction and context exposures was counterbalanced between animals). The day after the last extinction training, the fear renewal test took place, where rats were placed in context A for 6 min, where after a 300 s baseline, a CS was presented. For the 12 animals that received the injection of the control virus and 21 rats that received the injection of the ChR2 carrying virus, light stimulation was on for 300 s (with 20 Hz pulses) during the baseline before the CS presentation. A second control group was composed of 12 rats that received the injection of ChR2 but did not receive any optogenetic stimulation during the fear renewal test.

### 2.6 Context Fear Protocol

The rats underwent contextual fear conditioning in context A (which was chamber α for half of the animals and chamber β for the other half) for 12 min during which they received three foot‐shocks without coupling with any other stimulus at 300, 512, and 724 s after the initiation of the control programme, which was activated immediately following the placement of the animal in the chamber. The following day they were exposed to context B (chamber β for those who underwent conditioning in α, and chamber α for the others) for 25 min. On the third day of the protocol, the animals were placed again in context A for 6 min to test their context fear, and 26 rats received laser stimulation for the first 240 s (12 were injected with ChR2 and 14 with the control virus). Ten rats injected with ChR2 did not receive any photic stimulation during the context fear test.

### 2.7 Ambulatory Distance Protocol

To ensure that the observed behavioural effects were not due to nonspecific motor impairments, a subset of rats was tested in a 50 × 50 cm darkened open field (Med Associates) for a 9 min session while tethered by a patch cable connected to a rotary joint (Doric Lenses) positioned above the arena, which allowed rats to move freely in the environment. After a baseline of 180 s, 22 rats (13 who received ChR injection and 9 with the control GFP virus) received light stimulation for 180 s. 12 rats with ChR2 did not receive any light stimulation. Activity data were collected with an automatic system (Activity Monitor, SOF‐811, Med Associates), which estimated locomotor distance via infrared beam breaks. Total distance travelled was measured as a control to dissociate specific fear‐related effects from general motor effects of pathway stimulation.

### 2.8 Statistical Analysis

**FIGURE 3:**
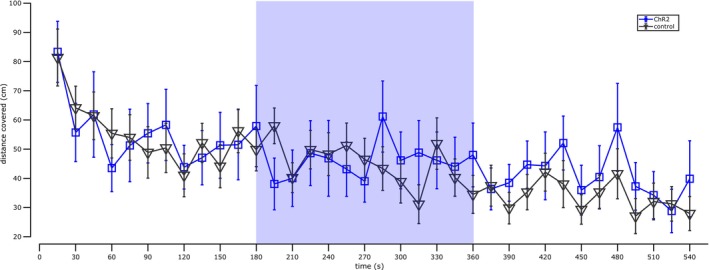
vHPC‐PL pathway stimulation does not affect locomotor behaviour in open field. Average distance travelled over time in an open field exploration test for laser‐stimulated animals expressing ChR2 (*n* = 13, blue line) and control animals (*n* = 21, black line). Shaded areas depict ±SEM, the shaded blue rectangle indicates when laser was ON. [No significant difference at any time point; unpaired *t*‐test, see Section 2 and Data S1]

Statistical analyses of behavioural experiments were performed with custom written programmes in Matlab (Mathworks, USA). For the fear renewal experiments (Figure 2a,c), we employed planned unpaired *t*‐tests comparing the groups specifically at the optogenetic stimulation time points and those right afterwards. This approach was chosen because no group differences were expected at other time points. For completeness, we note that no other time point showed a significant difference between groups, even without correction for multiple comparisons (see Data S1). For the fear renewal (Figure 2b) and ambulatory distance (Figure 3) experiments, we performed unpaired *t*‐tests at each time point without correction for multiple comparisons, to maximize sensitivity to any potential effect. Even under these liberal conditions, no significant group differences were observed at any time point (see Data S1). Overall, our experiments included two control groups: animals that received the injection of the control GFP virus and rats that received the injection of ChR2 but did not receive any light stimulation. No significant difference (unpaired *t*‐tests, *p* > 0.05) was detected at any time point of any experimental protocol between the two control groups, which therefore were pooled.

### 2.9 Histology

After all experiments, rats were deeply anaesthetized with a lethal dose of pentobarbital and intracardially perfused with saline (0.9%) followed by paraformaldehyde (4%). Coronal slices (35 μm) were cut with a cryostat, slide mounted, and imaged on an epifluorescence microscope. Slices were carefully analysed to verify electrodes and fibres placement in PL and for the expression of ChR2. Animals of both control and ChR2 groups displaying low levels of viral construct expression or misplacement of the electrodes/optic fibre were discarded from further analysis.

## 3 Results

To selectively control vHPC projections in the PL, we used an AAV to induce the expression of channelrhodopsin2 (ChR2) in vHPC neurons (Figure 1a). Histological analysis confirmed restricted infection in the ventral hippocampal formation (vHPC and ventral subiculum; Figure 1b). It also confirmed ChR2 expression in axon terminals within the PL ipsilateral to the injection site (Figure 1c), consistent with the known projection from the vHPC to the mPFC.

To validate our optogenetic approach and verify that stimulating these terminals actually induces an effect on downstream neurons in the PL, light pulses were delivered at 20 Hz in the PL of anaesthetized animals expressing ChR2 in vHPC terminals. Most units did not show any activity change with optogenetic stimulation; however, 71 out of 597 units (Figure 1e) displayed an electrophysiological response within 12 ms of the laser pulse onset. There were two principal response types (Figure 1d–f): initial excitation after the pulse onset or inhibition followed by an excitatory rebound at the end of the pulse. Overall, 11.8% of cells recorded in ChR2‐expressing animals significantly responded to light pulses (both excitatory and inhibitory responses), which was significantly more than chance (Figure 1f). Conversely, the proportion of significantly responding units in controls was not significantly different from chance levels and significantly lower than in ChR2 animals (Figure 1f).

To determine whether the activity of the vHPC to PL pathway during contextual processing before CS presentation plays a role in context‐dependent cued fear renewal, we conducted a first experiment using a compound renewal design. An ABC fear renewal protocol (fear conditioning in context A, extinction in context B, fear renewal test in context C) was followed by a second fear renewal test in the initial conditioning context A (Figure 2a, top). Optogenetic stimulation of the vHPC to PL pathway occurred during the baseline context exposure periods before CS presentation in the two fear renewal tests (both in C and A). This stimulation did not affect subsequent cued fear renewal in either context. However, vHPC‐PL optogenetic stimulation during the pre‐CS baseline period decreased contextual fear in the conditioning context A (Figure 2a, bottom), suggesting that vHPC to PL pathway activity may be involved in contextual fear expression.

To further explore this, we conducted an experiment where, after contextual fear conditioning, we optogenetically stimulated vHPC terminals in the PL during a contextual fear recall test. Rats received a mild foot shock in context A without the presentation of any discrete cue; then, after a one‐day interval, they were returned to the same chamber and tested for contextual fear recall (i.e., without CS presentation) while the vHPC to PL pathway was stimulated (Figure 2b, top). During the recall test, no behavioural differences were observed at any time point between animals expressing ChR2 at vHPC‐PL terminals who received laser stimulation during the recall test and control animals (Figure 2b, bottom). As this experiment indicated that vHPC‐PL activity did not affect contextual fear per se, we next hypothesized that vHPC‐PL pathway activity might control contextual fear expression only after some form of extinction training, similar to what occurs with cued fear expression (Szadzinska et al. 2021; Vasquez et al. 2019).

Our first experiment suggested that vHPC‐PL stimulation affects contextual fear in the conditioning context but not in a different one (Figure 2a), so we aimed to validate this result with a simpler ABA design (fear conditioning in context A, extinction in context B, and fear renewal test in context A; Figure 2c). The animals underwent auditory fear conditioning in chamber A, then auditory fear extinction in chamber B for 3–7 days until freezing to the CS was below 60%. Animals who did not meet this criterion by the 7th day were excluded from further analysis. Finally, the rats were re‐exposed to chamber A and the CS. During the fear renewal session, the laser was ON during the baseline context processing period before CS presentation, which decreased fear expression during this period in ChR2 animals relative to controls (Figure 2c). This confirmed our initial result, showing that optogenetic stimulation of the vHPC to PL pathway decreases contextual fear expression after extinction.

As a control, we tested whether this vHPC‐PL stimulation directly affects motor behaviour. Rats were allowed to forage in an open field, and no differences in locomotor behaviour were observed between stimulated animals and controls (Figure 3). These results indicate that the vHPC‐PL pathway can control contextual fear after cued‐extinction training.

## 4 Discussion

Previous research has shown that vHPC‐PL activity can regulate cued conditioned fear after extinction training (Vasquez et al. 2019; Szadzinska et al. 2021), but it was unclear whether vHPC‐PL could also control contextual fear. To test this, we stimulated vHPC neuron terminals in the PL during baseline context exposure in a post‐extinction fear renewal test, which led to a decrease in contextual fear. This pathway stimulation did not affect the animals' motricity, nor did it impact fear to the contexts themselves prior to extinction, demonstrating that the vHPC projection to PL can gate contextual fear expression after extinction learning. Here, the results suggest that the vHPC‐PL circuit exhibits selectivity for contextual fear expression when recall is preceded by a tone‐shock extinction procedure.

We performed a control electrophysiological experiment to validate our optogenetic approach, and these recordings showed that ~12% of mPFC neurons responded to vHPC terminal stimulation with either excitation or inhibition. This is expected, as vHPC terminals are known to contact both excitatory and inhibitory cells within the mPFC (Jay et al. 1992; Tierney et al. 2004). While only a subset of prefrontal neurons was entrained by vHPC stimulation, sparse neural activity can strongly influence behaviour (Houweling and Brecht 2008). We propose that stimulation during behaviour disrupted the temporal precision of vHPC–mPFC communication, interfering with the coordinated activity required for proper behavioural and cognitive control (Buzsáki 2010; Umbach et al. 2022). This may underlie the reduced contextual fear expression observed in our experiment. While our findings establish a causal role for vHPC–mPFC projections in modulating contextual fear behaviour post extinction, future studies incorporating in vivo electrophysiological recordings during behaviour will be essential to elucidate the underlying circuit dynamics and mechanisms.

Our observation that vHPC‐PL stimulation during the baseline pre‐CS period does not affect subsequent cued fear levels in a fear renewal test suggests that the effect reported by Vasquez et al. (2019) with prolonged pharmacogenetic excitation was likely due to increased vHPC‐PL activity during CS presentation, as also seen with optogenetic stimulation during CS (Szadzinska et al. 2021). These past results, together with our own showing a specific role of the vHCP‐PL pathway only after extinction, are consistent with previous findings demonstrating that vHPC inactivation induces an increase of PL activity only after extinction (Sotres‐Bayon et al. 2012), possibly because PL activity (which is important for fear expression; Vidal‐Gonzalez et al. 2006; Sierra‐Mercado et al. 2011) is at ceiling level before effective extinction (Sotres‐Bayon et al. 2012). One potential interpretation, compatible with current and previous findings, is that vHPC information flow to the PL may play a role in adjusting behavioural responses when the meaning of cues or contexts becomes ambiguous (Bouton 2002), as it is the case after extinction training conducted in a context different from the conditioning one. Disrupting vHPC activity, or its normal communication with the mPFC, may impair the ability of the latter to appropriately regulate fear behaviour.

Our aim was to directly investigate the effect of vHPC‐PL stimulation within the framework of fear renewal, which has specific clinical implications as a model for exposure‐based therapies of anxiety disorders. To this end, we explored the impact of vHPC‐PL stimulation on contextual fear within a fear renewal paradigm for cued fear, given that no fear renewal effect is possible after contextual extinction. The effect of the stimulation was specific for post‐extinction as it did not affect fear expression after contextual conditioning in the absence of extinction sessions, suggesting that the role of the vHPC‐PL pathway may be specific to modulating contextual fear after extinction. However, it is important to note that, beyond the presence or absence of extinction, our fear renewal and contextual fear conditioning experiments also differed in the use of background versus foreground contextual conditioning, with the presence or absence of a discrete auditory cue. These variations may influence how contextual information is processed and expressed. While such differences in task structure could contribute to the observed outcomes, they also reflect the diversity of conditions under which contextual fear is regulated. Our findings support a role for vHPC–mPFC projections in modulating contextual fear following extinction, though we acknowledge that task structure and cue competition may also influence this effect. Indeed, while one of our control experiments showed that vHPC‐PL stimulation does not affect contextual fear prior to extinction (Figure 2B), we did not address whether the vHPC‐PL also modulates contextual fear after the extinction of contextual fear itself, which future work should explore in more detail. It is important to note, however, that since fear levels are low after contextual fear extinction, a floor effect might obscure any freezing reduction effect due to vHPC‐PL stimulation.

Context‐dependent fear renewal is an effective protocol for modelling fear relapse after EBT in translational neuroscience. Understanding the mechanisms that regulate how contextual information is modulated after extinction training and EBT is crucial for increasing the effectiveness of these therapies for anxiety disorders. This has important implications for the treatment of anxiety disorders, as it uncovers a specific neural pathway that may be involved in the contextual modulation of fear processing, which in turn may play a key role in extinction learning and EBT.

## Author Contributions

**Marco N. Pompili:** data curation, formal analysis, investigation, methodology, project administration, software, visualization, writing – original draft, writing – review and editing. **Adam Eckmier:** conceptualization, data curation, investigation, methodology. **Ralitsa Todorova:** formal analysis, software, writing – review and editing. **Margot Tirole:** formal analysis, investigation. **Bill P. Godsil:** conceptualization, formal analysis, funding acquisition, investigation, methodology, project administration, supervision. **Thérèse M. Jay:** conceptualization, funding acquisition, project administration, supervision, writing – review and editing.

## Ethics Statement

All the experimental procedures were performed in accordance with institutional guidelines and the French national and European laws and policies, and approved by the Paris Descartes University Ethical Committee.

## Conflicts of Interest

The authors declare no conflicts of interest.

## Peer Review

The peer review history for this article is available at https://www.webofscience.com/api/gateway/wos/peer‐review/10.1111/ejn.70287.

## Supporting information


**Data S1:** Supporting Information.

## Data Availability

Upon publication acceptance, data will be available in the following repository: https://doi.org/10.5281/zenodo.17338990.
