# Supplementary material for: Ventral Hippocampus Modulates Prefrontal Control of Background Contextual Fear After Cued Extinction
Source: Eur J Neurosci. 2025 Oct 23;62(8):e70287. doi: 10.1111/ejn.70287 (PMC12548305; doi:10.1111/ejn.70287)
Supplement: Supplementary file 1 — Data S1: Supporting Information. [file EJN-62-0-s001.pdf]

	fear Conditioning baseline	fear Conditioning CS1	fear Conditioning CS2	fear Conditioning CS3	first Extinction baseline	first Extinction CS1-4	last Extinction baseline	last Extinction CS1-4	renewal C baseline	renewal C CS1-4	renewal A baseline	renewal A CS1-4
p	0.786	0.337	0.941	0.801	0.165	0.745	0.396	0.977	0.621	0.899	0.027	0.941
t	-0.28	-1.00	0.08	-0.26	1.48	-0.33	-0.88	0.03	0.51	0.13	2.51	0.08
df	12	12	12	12	12	12	12	12	12	12	12	12

Compound ABCA fear renewal

	fear Conditioning baseline	fear Conditioning ITI1	fear Conditioning ITI2	recall min1	recall min2	recall min3	recall min4	recall min5	recall min6
p	0.153	0.530	0.522	0.438	0.978	0.945	0.596	0.162	0.174
t	-1.47	-0.64	-0.65	0.79	0.03	0.07	-0.54	-1.44	-1.40
df	26	26	26	26	26	26	26	26	26

Contextual fear conditioning and recall test

	fear Conditioning baseline	fear Conditioning CS1	fear Conditioning CS2	fear Conditioning CS3	first Extinction baseline	first Extinction CS1-4	last Extinction baseline	last Extinction CS1-4	renewal baseline	renewal CS1
p	1.000	1.000	0.395	0.780	0.864	0.381	0.696	0.261	0.010	0.823
t	0.00	0.00	-0.87	0.28	0.17	-0.89	-0.40	1.16	2.80	0.23
df	26	26	25	20	18	25	15	20	24	22

ABA fear renewal

	0s	15s	30s	45s	60s	75s	90s	105s	120s	135s	150s	165s	180s	195s	210s	225s	240s	255s
p	0.897	0.497	0.976	0.347	0.845	0.638	0.586	0.804	0.653	0.581	0.725	0.577	0.065	0.984	0.921	0.918	0.511	0.492
t	-0.13	0.69	-0.03	0.95	0.20	-0.48	-0.55	-0.25	0.45	-0.56	0.36	-0.56	1.91	0.02	0.10	0.10	0.66	0.69
df	32	32	32	32	32	32	32	32	32	32	32	32	32	32	32	32	32	32

	270s	285s	300s	315s	330s	345s	360s	375s	390s	405s	420s	435s	450s	465s	480s	495s	510s	525s
p	0.195	0.550	0.152	0.675	0.745	0.263	0.918	0.315	0.353	0.861	0.272	0.479	0.648	0.329	0.315	0.833	0.800	0.351
t	-1.32	-0.60	-1.47	0.42	-0.33	-1.14	0.10	-1.02	-0.94	-0.18	-1.12	-0.72	-0.46	-0.99	-1.02	-0.21	0.25	-0.95
df	32	32	32	32	32	32	32	32	32	32	32	32	32	32	32	32	32	32

Open field exploration test
